# Rebmab200, a Humanized Monoclonal Antibody Targeting the Sodium Phosphate Transporter NaPi2b Displays Strong Immune Mediated Cytotoxicity against Cancer: A Novel Reagent for Targeted Antibody Therapy of Cancer

**DOI:** 10.1371/journal.pone.0070332

**Published:** 2013-07-31

**Authors:** Mariana Lopes dos Santos, Fernanda Perez Yeda, Lilian Rumi Tsuruta, Bruno Brasil Horta, Alécio A. Pimenta, Theri Leica Degaki, Ibere C. Soares, Maria Carolina Tuma, Oswaldo Keith Okamoto, Venancio A. F. Alves, Lloyd J. Old, Gerd Ritter, Ana Maria Moro

**Affiliations:** 1 Lab. de Biofármacos em Células Animais, Instituto Butantan, São Paulo, Brazil; 2 Recepta Biopharma, São Paulo, Brazil; 3 LIM14-Depto. de Patologia, Faculdade de Medicina, Universidade de São Paulo, São Paulo, Brazil; 4 Depto. de Genética e Biologia Evolutiva, Instituto de Biociências, Universidade de São Paulo, São Paulo, Brazil; 5 Ludwig Institute for Cancer Research, New York Branch at Memorial Sloan-Kettering Cancer Center, New York, United States of America; Center for Genomic Regulation, Spain

## Abstract

NaPi2b, a sodium-dependent phosphate transporter, is highly expressed in ovarian carcinomas and is recognized by the murine monoclonal antibody MX35. The antibody had shown excellent targeting to ovarian cancer in several early phase clinical trials but being murine the antibody's full therapeutic potential could not be explored. To overcome this impediment we developed a humanized antibody version named Rebmab200, expressed in human PER.C6® cells and cloned by limiting dilution. In order to select a clone with high therapeutic potential clones were characterized using a series of physicochemical assays, flow cytometry, real-time surface plasmon resonance, glycosylation analyses, immunohistochemistry, antibody-dependent cell-mediated cytotoxicity, complement-dependent-cytotoxicity assays and quantitative PCR. Comparative analyses of Rebmab200 and MX35 monoclonal antibodies demonstrated that the two antibodies had similar specificity for NaPi2b by flow cytometry with a panel of 30 cell lines and maintained similar kinetic parameters. Robust and high producer cell clones potentially suitable for use in manufacturing were obtained. Rebmab200 antibodies were assessed by immunohistochemistry using a large panel of tissues including human carcinomas of ovarian, lung, kidney and breast origin. An assessment of its binding towards 33 normal human organs was performed as well. Rebmab200 showed selected strong reactivity with the tested tumor types but little or no reactivity with the normal tissues tested confirming its potential for targeted therapeutics strategies. The remarkable cytotoxicity shown by Rebmab200 in OVCAR-3 cells is a significant addition to the traits of stability and productivity displayed by the top clones of Rebmab200. Antibody-dependent cell-mediated toxicity functionality was confirmed in repeated assays using cancer cell lines derived from ovary, kidney and lung as targets. To explore use of this antibody in clinical trials, GMP production of Rebmab200 has been initiated. As the next step of development, Phase I clinical trials are now planned for translation of Rebmab200 into the clinic.

## Introduction

Antibody therapy has been established as a powerful tool since early 20^th^ century with the use of passive immunotherapy against a diverse range of infectious diseases [1]. Following the coming of age of monoclonal antibody (mAb) technology, passive immunotherapy for cancer treatment has shown great clinical success encouraging a significant amount of research in the area [2]–[4]. Targeted therapy has proven the magic bullet idea conceived by Paul Ehrlich in the early 20^th^ century [5]. Target specificity, low toxicity and the ability to activate the immune system are some advantages of the therapeutic use of mAbs [6]. The major limitation for the therapeutic use of mAbs – immunogenicity caused by murine antibodies – was overcome by technologies to humanize mAbs, thus decreasing their murine characteristics. To date, about a dozen mAbs have been approved for the treatment of hematological malignancies and solid tumors [4]. Clinical efficacy of therapeutic antibodies for cancer treatment depends mainly on two types of antibody functional features: target-specific binding by the Fab (antigen binding fragment) domain and immune-mediated effector functions mediated by the Fc portion such as antibody-dependent cell mediated cytotoxicity (ADCC) or complement-mediated cytotoxicity (CDC) [7].

A promising mAb candidate for cancer immunotherapy – MX35 – was generated years ago from mice immunized with a cocktail of four human ovarian carcinoma cells at Memorial Sloan-Kettering Cancer Center. The MX35 mAb (mouse IgG_1_) showed homogeneous reactivity with approximately 90% of human ovarian epithelial cancers and a limited number of normal tissues by immunohistochemistry (IHC) [8]. The specific *in vivo* targeting and localization of MX35 to tumors was demonstrated using iodine-labeled MX35 in animal models of human ovarian cancer and subsequently also in patients with ovarian cancer. Using PET (positron emission tomography), MX35 uptake was measured and the ratios obtained for tumor to normal tissues were as high as 6∶1 in nude rats [9]. In nude mice, selective localization of MX35 was demonstrated after intra-peritoneal or intravenous injection with peak tumor to normal tissue localization ratios of 12∶1 and 14∶1, respectively [10]. In humans, biodistribution studies with ^125^I or ^131^I-labeled MX35 in 25 patients with advanced ovarian cancer showed proper targeting, with tumor to normal tissue uptake ratios ranging from 2.3∶1 to 34∶1 (mean 10.18∶1) [11]. Evaluation of radiolabeled MX35 F(ab')_2_ uptake in samples biopsied from patients with ovarian cancer revealed that MX35 localizes primarily to the micrometastatic ovarian carcinoma deposits within the peritoneal cavity [12]. Pre-clinical studies in an ovarian cancer model using MX35 labeled with either ^213^Bi or ^211^At demonstrated therapeutic efficacy when treating micrometastatic growths of the ovarian cancer cell line OVCAR-3 in mice [13], [14].

Initial immunochemical analyses described the MX35 antigen as a 95 kDa cell surface glycoprotein with a large protease-resistant region carrying the MX35 epitope [15]. The MX35 antigen has subsequently been identified as the sodium-dependent phosphate transport protein 2b (NaPi2b) encoded by the *SLC34A2* gene. NaPi2b is expressed on the surface of cancer cells as a heavily N-glycosylated protein with additional post-translational modifications and disulfide bridges in the major extracellular loop [16]. Overexpression of the *SLC34A2* gene and the NaPi2b protein was found in well-differentiated serous and endometrioid ovarian tumors [17], [18]. Immunohistochemical and mRNA expression data have further shown that two histologic subtypes of ovarian carcinoma express particularly high levels of NaPi2b: serous and clear cell adenocarcinomas. The former represents the most frequent subtype of ovarian carcinoma, while the latter is a rare subtype associated with poor prognosis [19]. While MX35 has been raised by the immunization of mice with ovarian carcinoma cells, and the antibody has been primarily explored for potential therapeutic application in ovarian cancer, the MX35 antigen is also substantially expressed in several other epithelial carcinomas, including lung, thyroid and renal cancers, potentially expanding the therapeutic application of the antibody to these and other types of cancer [16]. Altogether, pre-clinical and initial clinical data obtained with MX35 suggest this antibody as a potential therapeutic agent for treatment of cancer.

Due to the inherent immunogenicity of murine monoclonal antibodies in cancer patients their clinical use is highly limited and humanized antibodies are required for exploring the full therapeutic potential of mAbs. Here, we report on the construction, generation, and characterization of Rebmab200 mAb, a humanized version of the murine monoclonal antibody MX35. Humanization of Rebmab200 was achieved by using a veneering process for the murine VL and VH chains and subsequent engineering into a full length human IgG_1_ construct. The antibody was prepared in PER.C6 cells after stable transfection with the recombinant plasmid. Properties of the purified humanized antibody were compared side-by-side to mAb MX35 using a large series of biochemical, cell-biology and immunohistochemical assays. We confirmed that the humanization process did completely preserve the binding specificity and kinetics of the original mouse antibody. In addition to maintaining specific binding characteristics, Rebmab200 acquired desirable effector functions not previously present in the murine antibody, especially the capability for strong antibody-dependent cell-mediated cytotoxicity (ADCC) in *in vitro* assays with human cancer cells and human effector cells. Clinical grade of the humanized antibody Rebmab200 is now being produced for first clinical trials in cancer patients.

## Materials and Methods

### Ethics Statement

The IHC studies were conducted with the approval of the Medical Ethical Committee of the Clinics Hospital and Faculty of Medicine/University of São Paulo, Brazil. Human blood for cytotoxicity assays was supplied by the Blood Bank/Hemotherapy Institute of Hospital Alemão Hospital Oswaldo Cruz, São Paulo, Brazil.

### Cell Lines

PER.C6® cells (Crucell, Leiden, Netherlands), in adherent or suspension form, were maintained in DME-F12/FCS (Life Technologies, Carlsbad, CA, USA) or PERMAb medium (HyClone, Logan, UT, USA), respectively. The cancer and normal cell lines were obtained from Ludwig Institute for Cancer Research (New York Branch, NY), American Type Culture Collection (ATCC, Rockville, MD) or from our laboratory cell bank.

### Generation of the Rebmab200 expression vector

Humanized LC (light chain) and HC (heavy chain) sequences of the Rebmab200 variable domain were designed by the veneering method [20]. The human IgG_1_ constant region was inserted, and complete genes were synthesized by Geneart (Life Technologies) and cloned into pcDNA3002Neo [21].

### Cell Line Development

For a stable pool generation, adherent and suspension PER.C6® cells were transfected with pcDNA3002Neo/HC/LC/Rebmab200 using Lipofectamine (Life Technologies) and electroporation, respectively. Transfectants were selected by resistance to G418 (Sigma-Aldrich, St.Louis, MO, USA). Stable pools of cells were cloned by limiting dilution. Growing clones were selected by productivity. After transfer to shaker flasks, batch and fed-batch runs were performed. Samples were taken daily for VCD (viable cell density) measurement and antibody titer by RT-SPR (real-time surface plasmon resonance) using Biacore T100 (GE Healthcare, Uppsala, Sweden). Calculations included PDT (population doubling time), IVCC (integrated viable cell concentration) and Q_p_ (specific productivity). The stability of selected clones was monitored over 50 generations, during which VCD and titer were checked. At 4 time points during these runs, cells were taken to start a 10-day batch. Calculations were the same as described above.

### Purification of antibodies by protein A affinity

A HiTrap rProtein A FF column (GE Healthcare) was used to purify mAbs. Antibodies were eluted with a low pH buffer, neutralized and dialyzed against PBS. Antibody concentration was estimated by A_280_ (ε_280nm_ 207,360M^−1^cm^−1^; as calculated by ProtParam) [22].

### Determination of Rebmab200 titer by RT-SPR

The biotinylated epitope was immobilized on a SA biosensor chip. Clone 7 was defined as the Rebmab200 standard reference (SR). SR (ranging from 1 to 50 µg/mL) and diluted PER.C6® culture supernatants were injected over the sensor surface. A non-linear calibration curve (described by a 4-parameter function) was created to calculate sample concentrations.

### Flow Cytometry

First, 3×10^5^ normal or tumor cells were incubated with MX35 or Rebmab200 or an isotype matched humanized mAb unrelated to cancer (Zenapax, Roche). Anti-IgG-FITC (Sigma-Aldrich) was used as the secondary antibody. Cells were re-suspended in cell fixing solution for analysis with a Guava EasyCyte cytometer (Merck, Darmstadt, Germany).

### Immunohistochemistry

Slides with histological sections were deparaffinized and rehydrated. Heat-induced antigen retrieval was performed, endogenous peroxidase activity was quenched and endogenous biotin was blocked as well as non-specific protein-protein reactions. Slides were then incubated with biotinylated primary antibody, MX35 or Rebmab200, diluted 1∶50 and 1∶100, respectively. Reactions were amplified with streptavidin-horseradish peroxidase (Dako, Glostrup, Denmark) and signal was developed with DAB (Sigma-Aldrich), counterstained with Harris' hematoxylin, dehydrated and mounted with Entellan (Merck). Non-relevant immunoglobulin was used as negative control. All samples were assessed by two experimented pathologists. Positivity was defined at the cut-off point of 10% of the cells. Discrepancies were resolved by consensus re-assessment under a double-headed microscope.

### High precision kinetic analysis by RT-SPR

Biotinylated epitope and a nonspecific reference (synthetic peptides consisting of amino acids 311–339 and 189–216 of NaPi2b protein, respectively) [16] were immobilized on a SA sensor chip via streptavidin-biotin capture. Antibody solutions (5–80 nM range) and blanks (HBS-EP) were injected over the sensor surface followed by dissociation. Background was removed by subtracting the nonspecific reference surface response from response of the active surface. Double subtractive referencing was employed by subtracting the blank response. Kinetic constants were calculated by the global fitting method (1∶1 Langmuir binding model).

### Determination of mRNA levels

Total RNA was isolated from 1×10^7^ cells using an RNeasy Mini Kit with DNase-RNase-free (Qiagen, Hilden, Germany). First-strand cDNA was obtained using the SuperScript™ III First–Strand Synthesis System (Life Technologies). mRNA levels were analyzed with an Applied Biosystems 7500 Real-Time PCR system by the comparative Ct method [23] using GAPDH as a reference gene. PCR was performed using Power SYBR Green PCR Mastermix (Life Technologies). Specificity of amplification was determined by melting curve analysis.

### Glycosylation assay

N-linked glycans were released from proteins with PNGase F (Merck) and labeled with aminobenzamide. Maltose was used as an internal standard. Labeled glycans were separated by HPLC with a column derivatized with amido groups, utilizing an ammonium formate (250 mM)-acetonitrile gradient. Migration times were compared with those of a glucose homopolymer standard to assign glucose unit (GU) values.

### Cytotoxicity assays

ADCC and CDC (complement-mediated cytotoxicity) activities were assessed using a standard Chromium-51 release assay with cancer cell lines. In ADCC experiments, human peripheral blood mononuclear cells obtained from healthy donors were used as effector cells. When applicable, the Rebmab100 mAb (Recepta Biopharma, São Paulo, Brazil) was included as positive control. Results were expressed as the percentage of lysis. For CDC experiments, complement was obtained from healthy donors.

## Results

### Rebmab 200 maintains the binding properties of MX35

The recombinant humanized IgG_1_ antibody Rebmab200 is encoded by the fully synthetic and codon-optimized full-length plasmid pcDNA3002Neo/HC/LC/Rebmab200. For an initial characterization and to compare the recombinant antibody to its murine counterpart MX35, we expressed Rebmab200 in adherent growing PER.C6® cells after stable transfection and purified the recombinant humanized antibody from the culture supernatant of a stable clonal pool of antibody-expressing PER.C6® cells.

Both antibodies were co-typed by flow cytometry using a panel of cultured human cancer and normal cell lines to assess the capability of the antibodies to recognize the target antigen expressed on the cell surface of tumor cells. A total of 30 cell lines (NaPi2b expressing and NaPi2b non-expressing) were tested, of which 22 were tumor-derived cells (Table 1) and 8 were normal non-tumor cells (data not shown). The humanized antibody Rebmab200 and its murine origin MX35 completely co-typed by FACS and no binding differences were observed, including binding to the known NaPi2b-positive ovarian cancer cell lines OVCAR-3 and SK-OV-6, the renal cancer cell line SK-RC-18 and the lung cancer cell line SK-LC-1. Rebmab200 as well as MX35 did not bind to a series of cancer cell lines derived from cervix, prostate, colon, bladder, lung, stomach or melanoma, or to a panel of 8 normal cell lines derived from ovary, kidney, lung or retina, most of them known not to express NaPi2b.

**Table 1:** Immunoreactivity of MX35 and Rebmab200 in live cells by flow cytometry.

Tumor Cell Lines	Tissue source	MX35 Positive cells (%)/Median fluorescence	Rebmab 200 Positive cells (%)/Median fluorescence
SK-RC-18	Kidney	94.69/219.9	98.75/218.42
OVCAR-3	Ovary	95.71/182.62	94.64/173.97
SK-OV-6	Ovary	85.73/33.11	82.33/27.83
SW626	Ovary (metastasis of a primary colon tumor)	2.23/3.18	2.79/3.4
LoVo	Colon	3.53/2.88	2.63/2.61
DLD-1	Colon	1.96/3.03	1.92/2.98
SK-LC-1	Lung	91.43/36.05	90.81/34.46
H358	Lung	1.44/2.92	1.82/2.88
A549	Lung	1.64/2.88	2.96/3.33
CRL5802	Lung	0.21/2.59	0.55/2.62
NCI-1155	Lung	2.1/2.49	1.62/2.52
NCI-H23	Lung	0.74/2.13	0.84/2.15
LNCaP	Prostate	1.49/2.46	1.98/2.54
PC3	Prostate	0.46/2.85	0.32/2.72
DU145	Prostate	0.11/2.18	0.27/2.03
MCF-7	Breast	4.06/2.78	4.68/2.87
MDA-MB-231	Breast	1.21/2.59	0.55/2.56
SK-MEL-28	Skin	0.24/2.04	0.33/2.1
MKN45	Stomach	5.72/2.68	4.94/2.77
HeLa	Cervix	0.57/2.27	0.6/2.29
VMCub3	Bladder	1.02/2.81	0.93/3.01
5637	Bladder	0.43/2.55	0.33/2.19

**Figure 1:**
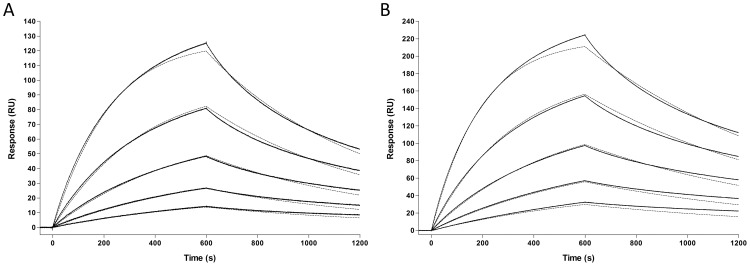
Global fitted (dotted line) SPR data of murine MX35 and Rebmab200 binding to immobilized synthetic NaPi2b epitope. MX35 (A) and Rebmab200 (B) were injected at concentrations ranging from 5 to 80 nM. After a 10 min association phase, the dissociation phase was followed for additional 10 min. Following double subtractive referencing, the curves were plotted using a 1∶1 Langmuir binding model, using Biacore T100 Evaluation Software. The solid line represents the experimental data and the dotted line the mathematical model for the binding of MX35 and Rebmab200 to the synthetic NaPi2b epitope.

In order to quantitatively compare the binding efficacy of MX35 and Rebmab200, we used RT-SPR. The kinetic parameters, *k*_a_ and *k*_d_, were determined for Rebmab200 and MX35 binding to a synthetic peptide derived from the previously established MX35 binding epitope (Figure 1). Three data sets were collected for both antibody-epitope interactions. The mean kinetic rate constants describing the Rebmab200-epitope interaction were calculated to be *k*_a_ (4.93±0.25)×10^4^M^−1^s^−1^ and *k*_d_ (1.18±0.17)×10^−3^ s^−1^, resulting in an equilibrium dissociation constant of *K*_D_ 23.84±3.18 nM at 25°C. The equilibrium dissociation constant of the MX35-epitope interaction was *K*_D_ 34.08±8.28 nM at 25°C, as the mean kinetic rate constants were calculated to be *k*_a_ (6.13±0.63)×10^4^M^−1^s^−1^ and *k*_d_ (2.12±0.72)×10^−3^ s^−1^. These kinetic data indicate that the complex formed between Rebmab200 and the epitope peptide is highly similar to that formed by MX35 and that humanization did not alter the binding kinetics.

### Rebmab200 mAb displays strong immune effector function

**Figure 2:**
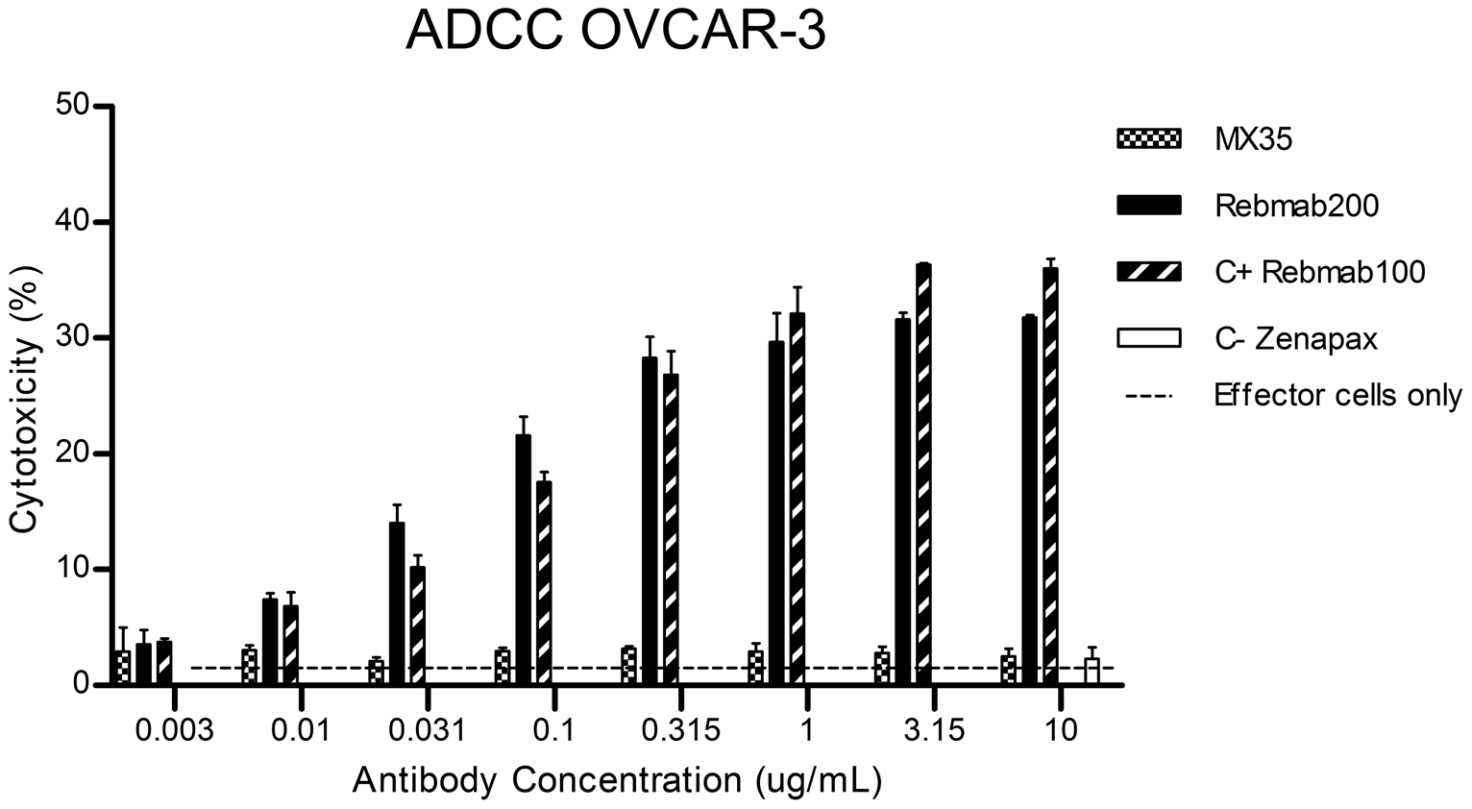
Comparison of ADCC activity between MX35 and Rebmab200 (stable pool) over a range of mAb concentrations. The % of cytotoxicity represents antibody-mediated cell lysis measured by release of ^51^Cr from labeled ovarian cancer cells (OVCAR-3). Effector cells were obtained from donated human peripheral blood. Rebmab100 was used as a positive control, and Zenapax (Roche) was used as a negative control. The assay was repeated with MCF-7 cells, a NaPi2b negative and Le^Y^ positive (Rebmab100 antigen) tumor cell line, showing no ADCC results (data not shown).

While MX35 was not able to mediate cytotoxicity towards NaPi2b-expressing cell lines by either ADCC or CDC, Rebmab200 displayed strong cytotoxicity by recruiting effector cells to lyse OVCAR-3 cells. The ADCC results for Rebmab200 were comparable to the ADCC results obtained with the anti-Lewis Y hu-mAb, Rebmab100 (Figure 2), demonstrated as being highly cytotoxic by both, ADCC and CDC [24]. *In vitro* ADCC activity of Rebmab200, but not of MX35, was not restricted to ovarian cancer cell lines but was also found with the renal cancer cell line SK-RC-18 (data not shown). Similarly to MX35, Rebmab200 did not display CDC activity for any of the tested tumor cell lines.

### Development of a Rebmab 200 production cell line

The promising results displayed by Rebmab200 purified from a stable pool of transfected PER.C6® cells prompted us to develop a production cell line suitable to generate material for pre-clinical and clinical studies. PER.C6® suspension cells were transfected with the vector pcDNA3002Neo/HC/LC/Rebmab200 and the recovered cells were cloned by limiting dilution, producing 210 clones. Based on the production titer in T-flasks a total of 32 clones were chosen for adaptation in stirred culture and subsequent fed-batch cultivation. Supernatants of the clones were evaluated by population doubling time, titer, length of run, density of viable cells and specific productivity. Clones were characterized by flow cytometry, RT-SPR, glycosylation profile and ADCC, in normalized concentration. Ten clones were further tested for long-term stability, with the goal of long-term production of the antibody through manufacturing. Although the continuous culture demonstrated a stable growth rate and productivity during a 50-generation period for the ten clones, batch runs starting at different time points of the continuous culture ruled out two of them. Physicochemical and biological assays were carried with mAb from all batches for each clone and the data generated was used to build a matrix for selection of the top 3 Rebmab200 clones.

### Characterization of Rebmab200 lead Clones: physicochemical, immunological and genetic properties

For further characterization of the selected Rebmab200 clones (namely, clone 45, 59 and 105), additional fed-batch runs were performed. Fed-batch cultures in 1 L vessels resulted in maximum cell density of 14 to 20 million cells/mL with antibody yields ranging between 2.3 and 3.5 g/L. A comparison of the three lead clones was performed through high precision kinetic analysis using RT-SPR. The affinity rate constants were evaluated, resulting in equilibrium dissociation constants (*K*_D_) in the same order of magnitude: *K*_D (clone 45)_ = 16.9 nM, *K*_D (clone 59)_ = 16.4 nM and *K*_D (clone 105)_ = 13.4 nM (data not shown).

**Figure 3:**
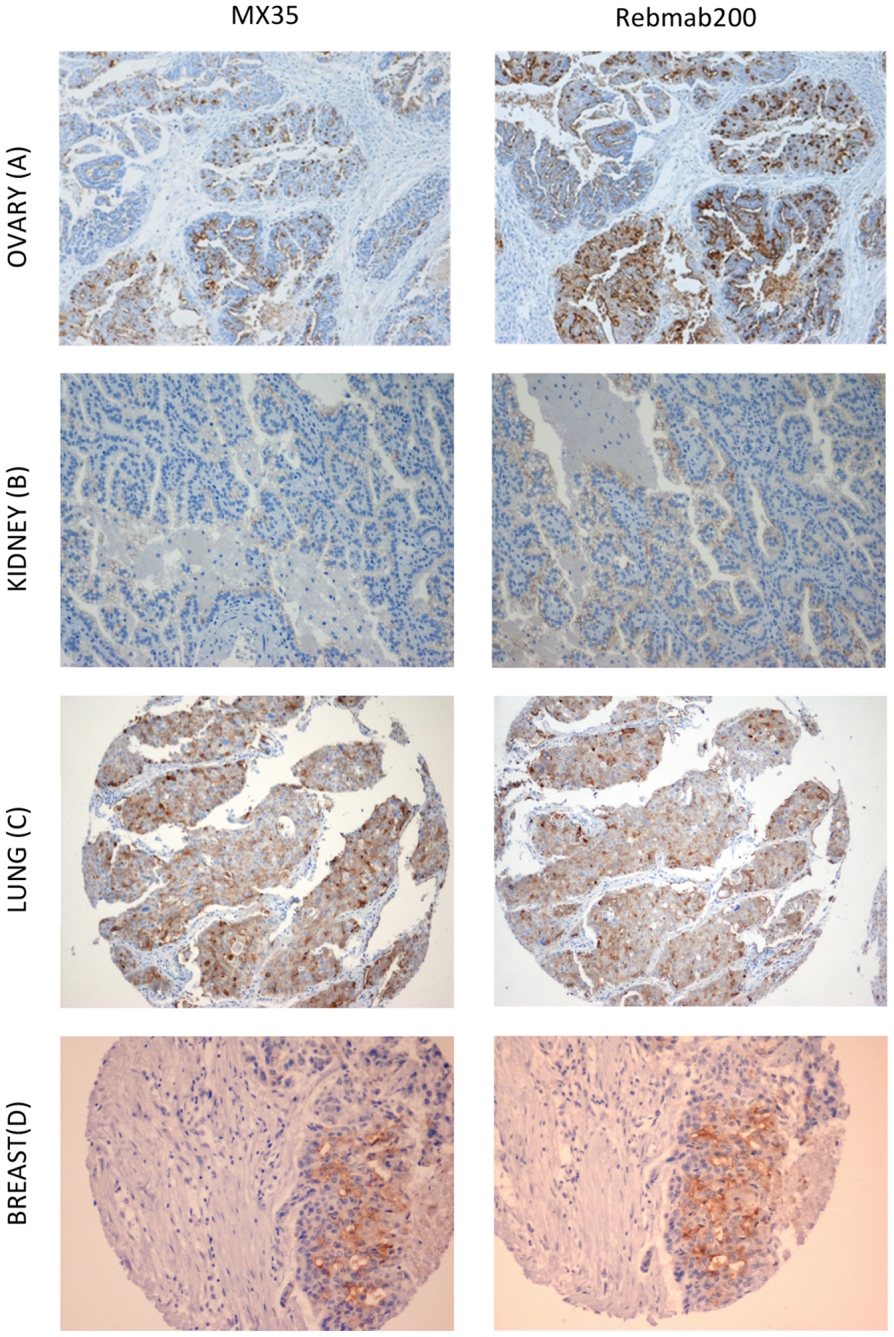
Comparison of the immuno-affinity of MX35 (left) at 1/50 dilution and Rebmab200 (right) at 1/100 dilution in parallel reactions using the same buffers and amplification and development conditions. Serial sections of serous papillary ovary adenocarcinoma (A), clear cell renal carcinoma (B), large cell poorly differentiated carcinoma of the lung (C) and invasive ductal carcinoma of the breast (D). Original magnification = 100×.

Immunological characterization of the mAb obtained from the three clones comprised IHC and ADCC to assess functionality of both, Fab and Fc portion of Rebmab200. The binding of MX35 and Rebmab200 to formalin-fixed parafin-embedded ovarian adenocarcinoma observed by IHC was assessed in a pilot study to set up the conditions. IHC revealed that Rebmab200, compared to MX35 yielded a stronger and more extensive reaction at cell membranes of ovary adenocarcinoma samples (Figure 3A) and similar results were obtained in all other organ specimen tested. Fifty ovarian carcinoma cases were tested (39 serous, 5 mucinous, 4 endometrioid and 2 clear cell types). Forty out of the 50 cases depicted membranous immunoreactivity for Rebmab200 (Table 2). Considering carcinomas of the kidney and lung, 18 out of 88 kidney adenocarcinomas and 56 out of 117 non-small cell pulmonary carcinomas showed membranous immunoreactivity for Rebmab200 in more than 10% of cells (Figure 3B and 3C, Table 2). Besides the expected binding to cancer tissues previously investigated during MX35 development, we also found invasive breast adenocarcinomas (ductal type) to show immunoreactivity with Rebmab200: 43 positive out of 177 specimens (Figure 3D, Table 2). The reactivity pattern of Rebmab200 was in accordance with the pattern displayed by MX35 (Table 2).

**Table 2:** Immunoreactivity of MX35 and Rebmab200 in tumor tissues by immunohistochemistry.

	Biotinylated MX35			Biotinylated Rebmab 200		
	n	positive	negative	n	positive	negative
Ovarian adenocarcinomas	50	40 (80%)	10 (20%)	50	40 (80%)	10 (20%)
Breast adenocarcinomas	177	53 (29.9%)	124 (70.1%)	177	63 (35.6%)	114 (64.4%)
Kidney adenocarcinomas	89	19 (21.3%)	70 (78.7%)	88	18 (20.5%)	70 (79.5%)
Lung non-small cell carcinomas	114	64 (56.1%)	50 (43.9%)	117	66 (56.4%)	51 (43.6%)

Among the 33 normal human organs required by FDA for assessment of biomarker distribution, fallopian tube, pancreas, breast, endometrium, uterine cervix, thyroid, kidney and lung more frequently displayed membrane immunoreactivity with Rebmab200. Low expression levels were found in liver, small bowel, peritoneum, appendix, prostate, thymus, and pituitary. Samples from all the other organs evaluated were negative for Rebmab200: brain, cerebellum, spinal cord, eye, bone marrow, lymph node, circulating blood cells, endothelium, heart, striated muscle, skin, esophagus, stomach, colon, spleen, parathyroid, adrenal, testis, placenta, ovary, bladder, and ureter.

**Figure 4:**
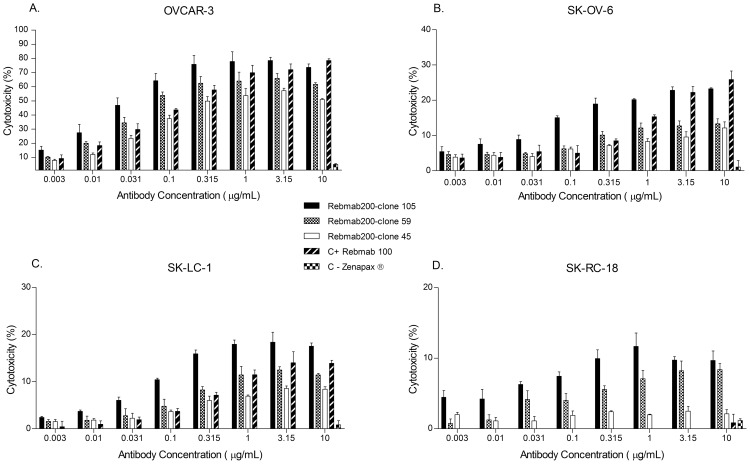
ADCC activity of Rebmab200 produced by the top three clones in two ovarian, OVCAR-3 (A) and SK-OV-6 (B), one lung, SK-LC-1 (C), and one renal, SK-RC-18 (D), cancer cell lines in comparison to Rebmab100. The % of cytotoxicity represents antibody-mediated cell lysis measured by release of ^51^Cr from labeled tumor cells. Effector cells were obtained from donated human blood. The assay was repeated with MCF-7 cells, a NaPi2b negative and Le^Y^ positive (Rebmab100 antigen) tumor cell line, showing no ADCC results (data not shown).

Evaluation of the immune effector function and potency of the three selected Rebmab200 clones was based on ADCC activity with two NaPi2b positive ovarian cancer cells lines, OVCAR-3 and SK-OV-6. Several antibody concentrations (Figure 4) and ratios of effector (E) to target (T) cells (data not shown) were used to determine dose-response curves for each clone, followed by a comparison among them. Although the Rebmab200 cytotoxicity levels varied in the analyzed cell lines, a consistent pattern was observed, that allowed ranking of the clones according to potency. Similar trends were seen for renal (SK-RC-18) and lung (SK-LC-1) cancer cell lines (Figure 4). Cytotoxicity levels of the clones were higher than those observed for the stable pool originally analyzed.

Effector functions are dependent on mAb glycosylation. To further support the clone selection, glycoforms were determined. Notwithstanding fucosylated forms were more abundant for all clones every one presented a degree of non-fucosylated glycoforms as well, with different ratios (data not shown).

**Figure 5:**
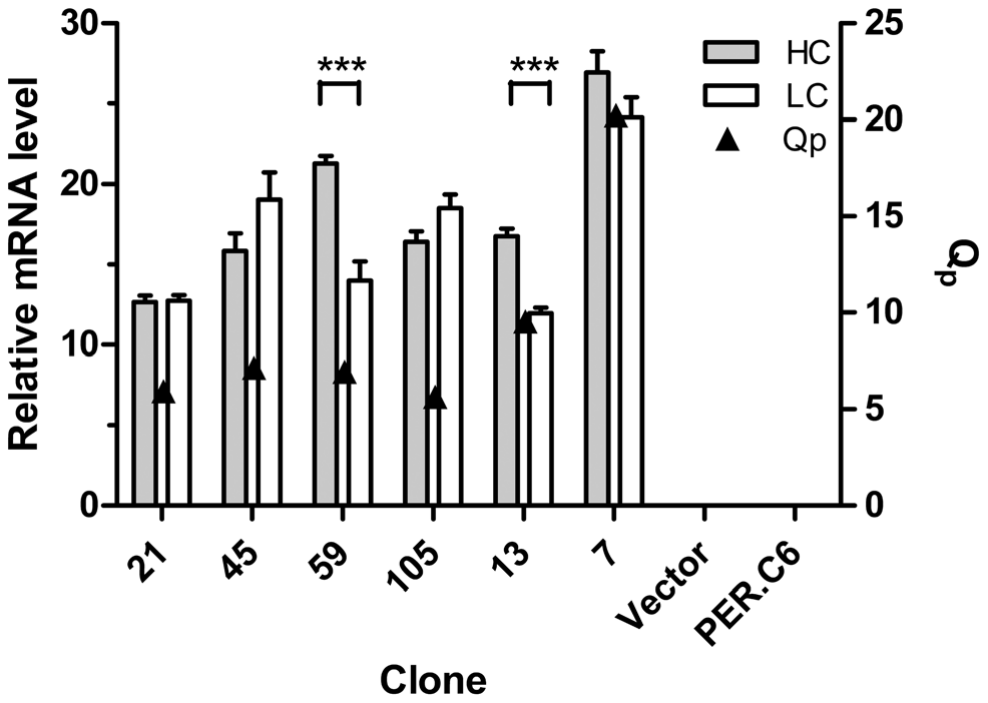
Immunoglobulin HC and LC mRNA levels and productivity of the most representative Rebmab200 clones as determined by RT-qPCR. **GAPDH was used as a reference gene.** Vector represents PER.C6®cells transfected with mock vector; PER.C6® corresponds to the parental cell. Error bars represent the standard deviation of triplicates. Qp represents specific productivity levels.

Immunoglobulin HC and LC mRNA levels and also specific productivity (Qp) of the most productive Rebmab200 clones were determined (Figure 5). Clone 7 (Rebmab200 SR) showed the highest mRNA levels, and small differences were observed among the other clones. The differences between HC and LC mRNA levels of clones 59 and 13 were statistically significant (p<0.05). Qp of the top Rebmab200 clones was similar. Immunoglobulin HC and LC gene copy numbers of the clones were also evaluated by qPCR. All clones contained one copy of each of the LC and HC genes. Parental cells and cells transfected with mock vector did not contain any copies of the antibody gene, as expected (data not shown).

## Discussion

Given the booming success with the use of mAbs for cancer treatment in recent years, we report the development and characterization of a novel antibody – Rebmab200 – with high potential for cancer immunotherapy. Rebmab200 is the humanized version of the murine monoclonal antibody MX35, which recognizes and targets the NaPi2b antigen, highly expressed in ovarian cancer and a series of other tumor types and with limited expression in normal tissues. Although radiolabelled MX35 was used in a limited number of cancer patients in the past and had shown adequate biodistribution and pharmacokinetics profiles in patients with ovarian cancer [11], [25], its therapeutic use was limited to single dosing due to its murine nature and the complications associated with long-term treatment with murine antibodies. Rebmab200 is potentially a better candidate for the treatment of ovarian and other types of cancer than MX35. This is not only because the antibody is humanized but also because of its additional cytotoxic immune effector functions which were not present in the original murine antibody MX35.

The affinity and the specificity of the humanized antibody for the antigen were fully retained when compared to its murine counterpart, and no significant changes have been observed in kinetic parameters and in the tissue binding profiles by immunohistochemistry. While MX35 presented a half-life for dissociation greater than 10 h [26], Rebmab200 forms a similar complex with the epitope as shown by its slightly lower equilibrium dissociation constant.

These results are encouraging, as often humanization of mAbs result in a loss in antigen binding properties in comparison to the original murine antibody [27]. It is well known that the MX35 antigen, NaPi2b, is involved in the transport of inorganic phosphate and the maintenance of phosphate homeostasis in the human body. Nevertheless, its function in tumor cells is yet not known. A study reported a minor decrease of NaPi2b-mediated phosphate transport in HEK293 cells transfected with NaPi2b after MX35 treatment, suggesting a potential inhibitory effect of MX35 on NaPi2b function [28]. We plan on conducting functional studies to evaluate function blocking capability of Rebmab200 and its subsequent effects on tumor cells.

Evaluation of the binding characteristics of the antibodies on a tumor cell line panel shown that many of the cell lines tested negative for antibody binding (murine or humanized), despite the high reactivity of the antibodies to human tumor tissues. A rather low frequency of NaPi2b expression in cultured tumor cell lines had actually been reported before. While MX35 antigen was detected in 16 out of 18 fresh ovarian carcinomas, its expression was rare in tissue cell lines, being detected in only 3 out of 8 ovarian carcinomas, 1 out of 8 lung carcinomas and 3 out of 11 renal carcinomas [8]. This discrepancy from the positivity obtained with fresh ovarian carcinomas compared to tissue cell lines was also observed in the present study with Rebmab200. It has been suggested that this antigen may be lost during adaptation of tumor cells to tissue culture [8]. Nevertheless, expression of the antigen in tumor tissues, identified by a large IHC panel including ovary, kidney, lung and breast tumors, attests its relevance as a therapeutic target. The results obtained with large sample sizes of distinct tumor tissues, derived from patients that can be treated, that is, displaying positive expression of NaPi2b reflected by binding of Rebmab200, are more relevant and support a clinical trial proposition with this antibody for targeted therapy for different types of cancer.

In breast cancer, a recent study analyzing *SLC34A2* gene expression in 146 breast tumor samples hypothesizes that this gene encoding NaPi2b antigen may be involved in the development of breast cancer and metastasis and suggests NaPi2b as a novel marker for detection of breast cancer and a target for therapeutic strategies [29].

Of particular importance for the clinical use of Rebmab 200 is its strong capability for immune effector function such as ADCC. Using *in vitro* ADCC assays the antibody was shown to mediate cytotoxicity in an antigen specific manner to tumor cells derived from different tumor types including ovarian cancer, renal cancer and lung cancer. In ADCC, an antibody first binds to its target on tumor cells, and following this, the Fc portion is recognized by Fc_γ_R on effector cells. Interaction of the Fc with the Fc_γ_R activates effector cells, resulting in the release of molecules contained in cytotoxic granules, such as perforin, granulysin and granzymes, which leads to lysis of tumor cells. The ability of therapeutic antibodies to induce ADCC depends on their binding affinity to both target and to the activating Fc_γ_R [7]. Rebmab200 has retained the affinity showed by MX35 mAb and acquired immune-effector function, which was evaluated at the clonal selection stage, allowing us to select clones with characteristics to support a desired mechanism of action. Immune-mediated tumor cell killing mechanisms (including ADCC, CDC and regulation of T cell function) and abrogation of tumor cell signaling are the most successful strategies in mAb-based targeted cancer therapy thus far, leading to approval by the FDA. ADCC is part of the mechanism of action of four (Trastuzumab, Cetuximab, Rituximab and Ofatumumab) of the eight naked antibodies currently approved for use in cancer treatment [4].

Like mAb MX35, Rebmab200 did not mediate cell killing through complement, despite the fact that the human IgG_1_ constant region of Rebmab200 contains all the elements found to be critical for complement fixation, Glu_318_ Lys_320_ Lys_322_ and Pro_331_ in CH_2_ region for C1_q_ binding [30], [31] and Ser-Thr-Ser in CH_1_ region for C3 binding [32]. In comparison to other human IgG_1_ constant regions, Rebmab200 presents two amino acids modifications in CH_3_ region, Asp for Glu and Leu for Met at positions 378 and 380, respectively. While these changes involve amino acids of the same nature and are not in close proximity to complement activation sites, one would not expect them to interfere with complement fixation. One of the explanations for this, however, may be related to the nature of the antigen recognized by the antibodies. NaPi2b is a large multi-passage transmembrane glycoprotein and the binding epitope may be too far from the actual cell surface for the complement factors to be inserted functionally into the cell membrane after being recruited by the antibody. Other antigens with complex nature have found to present resistance to complement lysis [33] leading to absence of CDC against cancer cells. The contribution of complement to anti-tumor activity of other antibodies in patients is not very clear, as resistance of cancer cells to complement-mediated lysis has been described, and the most common biological activities of antibodies currently in the clinic include ADCC, blockage of growth factor receptor signaling with subsequent apoptosis, and interference with angiogenesis pathways [34].

The remarkable cytotoxicity of Rebmab200 in our ADCC assay using OVCAR-3 cells as target cells is particularly interesting. The OVCAR-3 cell line was established from malignant ascites of a patient with progressive adenocarcinoma of the ovary after combination chemotherapy with cyclophosphamide, adriamycin and cisplatin. Consistent with its origin, *in vitro* experiments demonstrated that OVCAR-3 cells indeed show resistance to clinically relevant concentrations of adriamycin (5×10^−8^M), melphalan (5×10^−6^M) and cisplatin (5×10^−7^M) [35]. The cytotoxicity caused by Rebmab200 in these cells suggests the potential activity of this antibody against chemoresistant ovarian tumors. In fact, recently, NaPi2b was found to be highly expressed in clear cell carcinoma [19], a subtype of ovarian cancer chemoresistant to platinum-based regimens, thus reinforcing the applicability of Rebmab200 to treat patients who do not respond well to classic chemotherapy.

To evaluate the magnitude of the immune-effector potential of Rebmab200, we included a potent cytotoxic antibody – Rebmab100, as a control reagent in the ADCC assay. This antibody recognizes the LeY blood group antigen found in membrane glycolipids and glycoproteins and was considered one of the most potent mediators of cytotoxicity to cancer cells [36]. Rebmab200 showed similar or greater cytotoxic activity towards ovarian and lung tumor cells in comparison to Rebmab100. So far the potent immune-effector functions (ADCC and CDC) of Rebmab100 might be the main mechanism of action of this mAb on tumor cells [24], [37], and are likely sufficient for its therapeutic potency. Safety and desirable pharmacokinetic profiles of Rebmab100 were demonstrated in a Phase I clinical trial in patients with epithelial carcinomas [37] and promising results have been obtained in a Phase II clinical trial conducted in Brazil [38]. Altogether, the similar potent immune effector function of these two mAbs supports the potential efficacy of Rebmab200 as a novel cancer therapy agent.

To allow evaluation of the newly developed antibody Rebmab200 in clinical trials we have embarked on GMP compliant production cell line development and downstream processing. Production cell lines were developed from PERC.6® cells after stable transfection with the humanized antibody gene construct. PERC.6® cells proved to be a robust production platform for the recombinant Rebmab200, with yields reaching 3.5 g/L before any further process development. This outcome is not related to high copy numbers being inserted into the host genome, as demonstrated here, which could lead to clone instability [39]. Because the PERC.6® cells are a human cell line carrying the same glycosylation patterns as those found in human blood, immunogenicity risks are reduced. Moreover, human glycosylation patterns favor recognition of the antibody by human immune effector cells, what might potentially increase its therapeutic value. The antibody is currently being produced under GMP conditions. We propose Rebmab200 as a novel targeted agent for cancer therapy, with high potential for treatment of ovarian, renal, lung and breast carcinoma. Phase I clinical trials are planned for translation into the clinic as the next step of development.
